# *Ureaplasma urealyticum* infection following organ transplantation: a case report and narrative review

**DOI:** 10.1080/0886022X.2024.2395466

**Published:** 2024-08-27

**Authors:** Hongru Zhang, Liping Yang

**Affiliations:** a Department of Pharmacy, ZhangJiakou First Hospital, Zhangjiakou, Hebei Province, China; b Department of Pharmacy, Beijing Hospital, National Center of Gerontology, Institute of Geriatric Medicine, Chinese Academy of Medical Sciences, Beijing Key Laboratory of Assessment of Clinical Drugs Risk and Individual Application (Beijing Hospital), Beijing, China

**Keywords:** *Ureaplasma*, kidney transplantation, hyperammonaemia, infection, organ transplantation

## Abstract

**Objective:**

One case of *Ureaplasma urealyticum* (UU) infection after kidney transplantation was reported, and relevant literature was collected to provide a scientific reference basis for clinical diagnosis and treatment.

**Methods:**

A case of UU infection after renal transplantation in our hospital was analyzed retrospectively. PubMed, Embase and Cochrane databases were searched for case reports of UU infection after organ transplantation before 30 June 2024. The clinical and laboratory characteristics, treatment and prognosis of UU infection were summarized and analyzed.

**Results:**

A 65-year-old man underwent renal transplantation on 26 January 2022 due to chronic renal disease (grade 2) caused by focal sclerosing glomerulonephritis. Hyperammonaemia and coma occurred after the operation, and the patient died. A total of 38 case reports or series of cases were included in this study, involving 44 patients. The case reports included 22 cases of kidney transplantation, 11 cases of lung transplantation, 4 cases of heart transplantation,1 case of liver transplantation and 6 cases of multiple organ transplantation. *Ureaplasma urealyticum* infection occurred in 74.47% of cases within 1 month after transplantation, and the main symptoms after the infection were mental. After the onset of the disease, the most abnormal examination index was the increase of blood ammonia, followed by the increase of white blood cells. Therapeutic drugs included tetracyclines (doxycycline or minocycline), quinolones and azithromycin. The clinical symptoms could be significantly improved after 24 h of taking the fastest-acting medication. The highest mortality rate was in patients infected with *Ureaplasma* after lung transplantation.

**Conclusion:**

Early identification of UU and timely and correct drug treatment are essential to saving the lives of patients.

## 1. Introduction

*Ureaplasma urealyticum* (UU) is a member of the *Mycoplasma* family, which mainly lives on the mucosal surface of humans and various animals in the form of extracellular parasites. About 25%–40% of men and 80% of women have asymptomatic UU colonization in the urinary tract [1]. Therefore, the role of this organism in human diseases has been difficult to determine. It is considered a possible cause of various urinary diseases, including nonspecific urethritis, pelvic inflammation and chorioamnionitis. In patients with immunodeficiency, UU is more predisposed to causing disseminated infection due to the impaired defence function of the immune system. This heightened susceptibility is particularly associated with the development of hyperammonaemia syndrome (HS) and ascites [2]. One study reported the first case of HS caused by fluoroquinolone-resistant *Ureaplasma* strains and found that two empirical antibiotics could be used in severe *Ureaplasma* infections, particularly in patients with immunodeficiency, due to potential antimicrobial resistance [3]. Ranganath et al. discovered that polymerase chain reaction (PCR) analysis, along with microbial cell-free DNA next-generation sequencing (NGS) detection (known as the Karius test), could potentially emerge as a novel tool for augmenting the diagnosis of disseminated UU infection, thereby enhancing diagnostic accuracy [4].

After searching relevant literature, a few reviews related to UU infection after organ transplantation have been found, and most studies have focused on case reports or reviews of certain organ transplants without comprehensive review. By analyzing the clinical data of a case of UU infection after renal transplantation and collecting the relevant literature, this study explores and summarizes the clinical features and treatment methods of UU infection after various organ transplants. The goal is to help the early and accurate diagnosis and treatment of UU.

## 2. Case report

**Figure 1.**
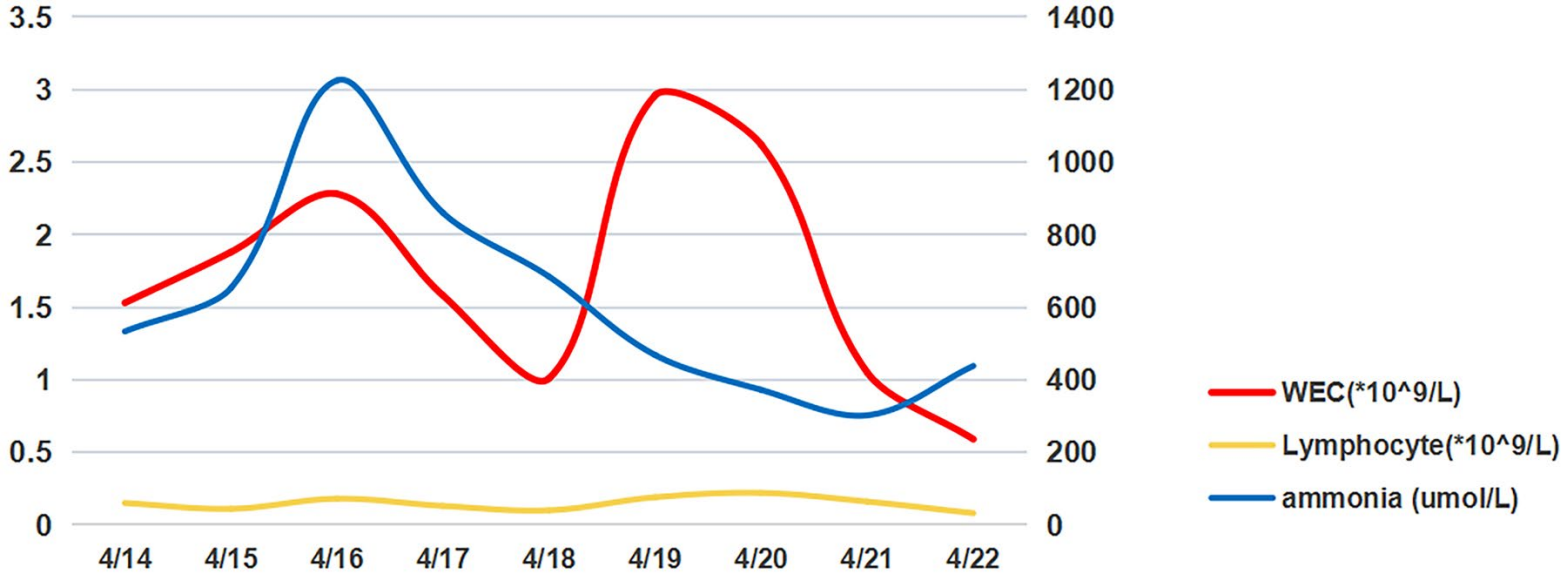
WBC, Ammonia, and lymphocyte levels of patient from 1 to 9 days in hospital. WBC: white blood cell.

A 65-year-old man underwent renal transplantation in our hospital on 26 January 2022 due to chronic renal failure caused by focal segmental glomerulosclerosis. Mycophenolate sodium enteric-coated tablets and cyclosporine capsules were administered postoperatively to prevent rejection. Valganciclovir tablets were provided for antiviral therapy, and the patient was discharged once renal allograft function had returned to normal. In March 2022, the patient developed intermittent tolerable pain in both lower limbs. A deep vein ultrasound revealed deep venous thrombosis in the right lower limb. The patient was prescribed aspirin, clopidogrel for antiplatelet therapy and enoxaparin for anticoagulation. Since 11 April, the patient felt aggravation in both lower limbs, accompanied by systemic pain and severe neck pain for which he took painkillers. He then developed anorexia, nausea and vomiting, and the vomitus was mostly gastric juice. The patient had poor sleep quality and decreased activity. On the afternoon of 13 April, the patient’s family noticed symptoms of apathy and dullness, prompting them to call for an ambulance to bring the patient to our hospital for treatment. The patient lost consciousness upon arrival at the emergency department but responded to calls. Subsequently, the patient was promptly intubated and transferred to the intensive care unit for further treatment. On admission, the cyclosporine A. level detected was 177.50 ng/mL (normal range is 100–400 ng/mL), the number of white blood cells (WBCs) was 1.53 × 10^9^/L, the proportion of neutrophils was 91.4%, C-reactive protein (CRP) was 416.34 mg/L and procalcitonin (PCT) was 0.72 ng/ml, indicating an unexplained infection with low immune status. The patient received a regimen consisting of 2 g of ceftriaxone intravenously once daily, 600 mg of linezolid injection intravenously every 12 h as an anti-staphylococcal agent and 0.2 g of ganciclovir intravenously once daily for antiviral therapy. Additionally, the patient was prescribed 720 mg of mycophenolate sodium enteric-coated tablets *via* nasogastric tube every 12 h, 150 mg of cyclosporine capsules *via* nasogastric tube every 12 h to suppress immune cells and 0.4 mL of nadroparin calcium injection subcutaneously every 12 h for anticoagulation therapy. Further treatment included 16 mg of methylprednisolone tablets *via* nasogastric tube once daily, 40 mg of omeprazole sodium injection intravenously once daily for symptomatic relief and 47.5 mg of metoprolol succinate tablets *via* nasogastric tube once daily. Maintenance of previous medication was ensured with 20 mg of atorvastatin calcium tablets *via* nasogastric tube once daily. The patient received bedside continuous hemofiltration with an applied dose of 35 mL/kg/hour and a duration of 48 h, lactulose given by nasogastric tube to lower blood ammonia levels, cisatracurium besylate to manage seizures, mannitol (125 mL every 8 h for 2 days) for cerebral edema treatment and an amiodarone micropump (0.5 mg/min) to regulate heart rhythm (supraventricular tachycardia). Serum osmolality was monitored during CRRT using a microosmometer (Model 3300, USA). On the 4th day after admission, blood ammonia decreased to 860 μ mol/L (see Figure 1). On the 5th day of admission, the second-generation sequencing of blood and cerebrospinal fluid showed that Epstein–Barr virus was detected in both blood and cerebrospinal fluid, with copy numbers of 237 and 156, respectively. In addition, cytomegalovirus, torque teno virus and UU were also detected in the blood (the copy number of *Ureaplasma* was 420, whereas minicircular virus levels were below 100). Levofloxacin injection (0.5 g intravenously once daily) was given to the patient as an anti-mycoplasma treatment. Due to prolonged QT interval following medication, minocycline capsules (100 mg every 12 h) were administered *via* nasogastric feeding. Ganciclovir dosage was adjusted to 0.5 g every 12 h, and mycophenolate sodium enteric-coated capsules were halved. Prophylactic ornithine aspartate and reduced glutathione were administered to protect the liver. On the 8th day of admission, blood ammonia levels were measured at 302 μmol/L. Brain computed tomography (CT) revealed a homogeneous appearance of the entire brain, extensive brain tissue swelling, loss of normal sulci and cerebellar gyrus ventricles and signs suggestive of widespread subarachnoid hemorrhage. Consistent with hypoxic-ischemic encephalopathy, there was a high probability of brain death. On the 9th day of admission, blood ammonia levels were recorded at 438 μmol/L (see Table 1). A head CT scan revealed diffuse brain edema, decreased density of brain parenchyma and subcutaneous soft tissue swelling extending from the top to the left side. An abdominal CT scan demonstrated renal transplant-related atrophy in both kidneys, multiple cysts, exudative changes surrounding both kidneys and multiple pelvic effusions or exudative changes. The patient developed haemodynamic instability, carbon dioxide retention and poor vital signs. Finally, the patient was determined to be brain dead by an organ donation agency.

**Table 1.** Various examination indicators of patients in our hospital.

The course of days	The references ranges	4/14	4/15	4/16	4/17	4/18	4/19	4/20	4/21	4/22
WBC(*10^9／L)	4.0-10.0	1.53	1.88	2.28	1.58	1.01	2.96	2.62	1.05	0.59
NEUT(%)	50-70	91.4	84. 1	86.0	86.7	83.2	92.5	93.2	88.5	86.4
CRP(mg/L)	0.068-8.2	416.34	>370. 14	355.74	364.0		407.22	343.59	311. 11	406.92
PCT(ng/ml)	<0.05	0.72					3. 12	2. 10	3.02	8.36
Ammonia (umol/L)	33-83	533	653	1225	860	684	468	372	302	438
BNP(pg/ml)	0-100	482.4	251.82	408.55	482. 13	984.27	2041.83	2045.62	1686.69	3774.23
TNI(pg/ml)	<40	185	105.3	116.4	105.0	91	94.6	77.5	99.9	77.3

WBC: white blood cell; NEUT: neutrophil; CRP: C-reaction protein; PCT: Procalcitonin; BNP: Brain natriuretic peptide; TNI: Troponin I.

## 3. Methods

### 3.1. Information sources

PubMed, Embase and Cochrane databases were searched using the following keywords: *Urealyticum*, transplantation, organ transplantation and *Ureaplasma urealyticum* to research relevant literature on UU infection after organ transplantation before 30 June 2024.

### 3.2. Literature inclusion and exclusion criteria

The inclusion criteria were as follows: case reports or case series with detailed clinical data and extractable complete case information.

The exclusion criteria were as follows: repeated reports, animal experiments, clinical trials and secondary analysis of literature; secondary infection or death caused by other factors; literature in which the original text or full text could not be found; incomplete medical history information of reported cases.

### 3.3. Literature screening

**Figure 2.**
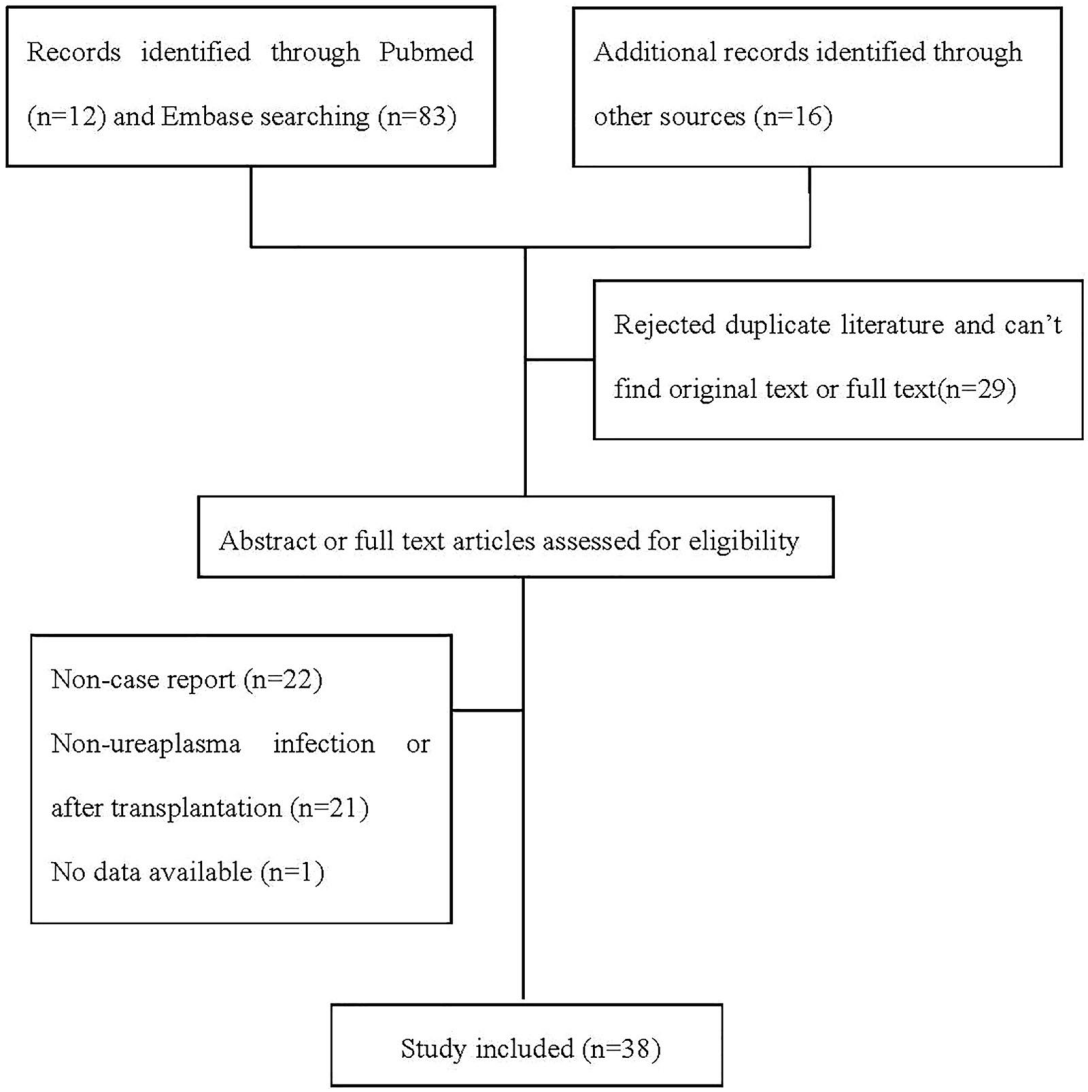
Flow chart describing selection process of the references.

Cases in the literature were carefully screened and assessed according to the inclusion and exclusion criteria. References were checked to ensure the accuracy and completeness of the case data following a review of the full article (the detailed process is shown in Graphical Abstract Figure 2). A total of 38 case reports or case series involving 43 patients were finally included in this study (see Table 2).

**Table 2a.** Summary of review cases Information of the liver transplantation.

Trans organ	Author/year	Gender	Age	Symptom	Time post transplant	Anomaly index	Drug /course	Prognosis
Liver	Haller, M. 1991 [32]	Female	49	Purulent secretion of the abdominal wall	12d	Leucocytosis 37000 cells/gL	Ciprofloxacin 400 mg/d; 3d	Recovered

**Table 2b.** Summary of review cases Information of the kidney transplantation.

Trans organ	Author/year	Gender	Age	Symptom	Time post transplant	Anomaly index	Drug /course	Prognosis
Kidney	Dolan, M.A. 2021 [33]	Female	61	Infected hematoma and persistent leukocytosis	8 d	A large right retroperitoneal and perinephric hematoma	Doxycycline (100 mg, bid) and moxifloxacin (400 mg bid); 45 d	Recovered
Kidney	Cheema, F. 2020 [2]	Female	56	A fever of 102 °F, acute onset of confusion, and abdominal pain	10 d	Serum creatinine and BUN to 5.7 and 102 mg/dL, leukocytosis of 18.2 × 103 /L with 98% neutrophils	Moxifloxacin 400 mg qd and doxycycline 100 mg bid; 21d	Recovered
Kidney	Higgins, A.B. 2020 [3]	Female	16	Altered mentation, blurred vision, asterixis, and worsening left knee arthritis	24 months	Serum ammonia >600 μmol/L	Azithromycin and doxycycline for 2 weeks	Recovered
Kidney	Adams, M. 2020 [34]	Male	42	Nausea, malaise, and pain at his kidney graft site	4months	Abscess formation anterior to the grafted kidney	Moxifloxacin (2w) and doxycycline (12w)	Recovered
Kidney	Legouy, C. 2020 [35]	Female	65	Refractory status epilepticus	3 months	Serum ammonia 671 μm ol/L	Levofloxacin (500 mg, qd) and doxycycline (200 mg, bid); 4 w	Died
Kidney	Kanipakam, R. 2020 [36]	Female	18	Fevers, fatigue and multiple joint pains; confusion, gait instability and blurry vision	Not mentioned	Creatinine up at 2.58 mg/dl from a baseline of 1.8 mg/dL, serum ammonia level was 600 umol/L	Levofloxacin and Doxycycline; 6 weeks	Recovered
Kidney	Devine, P. A. 2019 [37]	Female	55	Visible hematuria	18 months	Serum creatinine 3.61 mg/dL. CRP 72.7 mg/l, hemoglobin of 9.3 g/dl, and white cell count of 2.0*103/ml	Doxycycline	Recovered
Kidney	Kiberenge, R. K.2015 [38]	Male	35	Nausea, vomiting, and altered mental status	9 d	Ammonia level of 646 μmol/L repeat level 3 h later was 939 μmol/L	CRRT, sodium phenylbutyrate, and sodium benzoate	Died
Kidney	Geissdorfer, W. 2008 [39]	Male	38	Meningitis	4w		Doxycycline (200 mg q24h) and moxifloxacin (400 mg q24h, replaced by chloramphenicol (1 g q6h)); 48h	Recovered
Kidney	Loupy, A. 2008 [40]	Male	68	Decrease of the urinary flow, low-grade fever	19d	WBC count was 12 200/mm3, and the CRP level rose to 228 mg/L	Moxifloxacin (400 mg/day); 17 days	Recovered
Kidney	Loupy, A. 2008 [40]	Female	55	Fever and a perinephric collection	25d		Levofloxacin	Recovered
Kidney	Loupy, A. 2008 [40]	Female	52	Urinary leak associated with fever	4d	Two perinephric collections, and the urinalysis showed aseptic leucocyturia	Levofloxacin	Recovered
Kidney	Eilers, E. 2007 [41]	Female	19	Abdominal pain, dysuria, macrohematuri, loss of weight and general fatigue	8 yaers	Serum creatinine level 1.63 mg/dl, WBC 1,000 /L, multiple abscesses of kidney	Levofloxacin (10 mg/kg/day); 8w	Recovered
Kidney	Camara, B. 2007 [42]	Female	29	Perikidney allograft pain and fever (38 °C)	4 m	CRP 209.528 nmol/L; WBC 9.8 × 109/L	Imipenem(2g/d) and gentamicin (3 mg/kg/dfor3day s), peritonealcavity; 3 m	Recovered
Kidney	Cordtz, J. 2006 [43]	Female	35	Tenderness, erythema and swelling around the left ankle, temperature was 37.0 C	8 years	CRP was 51 mg/L, blood LKC 3. 1*109 /L, plasma urate 0.67 mmol/l, and P-CREA 0. 187 mmol/l.	Clarithromycin 500 mg b.i.d. and doxycycline 100 mg b.i.d; 6.5 weeks	Recovered
Kidney	Mian, A. N. 2005 [44]	Female	15	Right hip flexor weakness and leg pain	6 w	WBC4700/mm3 (83% polymorphonucl ear cells, 8% band forms), erythrocyte sedimentation rate 69 mm/h	Doxycycline and levofloxacin; 2 m	Recovered
Kidney	Pastural, M. 2002 [7]	Male	30	Abdominal tenderness but no fever and no sign of wound infection	Immediate	Leucocyturia (2*105 cells/ml)	Ofloxacin; 12d	Recovered
Kidney	Souweine, B. 1998 [45]	Female	48	Ureteral necrosis, fever	3w	Intrapelvic and wound abscesses	Quinupristin/dalfo pristin, 350 mg q8h for 15 days; streptogramins for 36 days	Recovered
Kidney	Miranda, C. 1990 [46]	Male	37	Developed a high fever	Immediate	Pus-filled perikidney abscess	Doxycycline, 100 mg every 12 hr	Recovered
Kidney	Miranda, C. 1990 [46]	Female	22	Acute kidney failure with oliguria and a fever of 38 °C	Immediate		Oral doxycycline,100 mg every 12 hr	Recovered
Kidney	Miranda, C. 1990 [46]	Female	55	An open, oozing surgical wound with a purulent, but not foul-smelling, exudate and a fever of 38 °C	20d		Doxycycline, 100 mg every 12 hr; temperature to normal within 48 hr	Recovered
Kidney	J. E.MOKHBAT, 1982 [47]	Male	31	Fever, abdominal pain, ileus, nd wound drainage	6 d		Minocycline; 6w	Recovered

**Table 2c.** Summary of review cases Information of the lung transplantation.

Trans organ	Author/year	Gender	Age	Symptom	Time post transplant	Anomaly index	Drug /course	Prognosis
Lung	Sankhesara, D. M.2022 [24]	Male	65	Hallucinations	6 d	Serum ammonia (NH3) was 549 µmol/L	Moxifloxacin, azithromycin and dialysis; 7 d	Recovered
Lung	Shrestha, K.2022 [48]	Female	30	Decreased responsiveness	7 d	ammonia levels 353 mmol/l	CRRT, lactulose, polyethylene glycol, levocarnitine, rifaximin, acarbose &sodium benzoate	Died
Lung	Chan, P.G.2021 [17]	Male	34	Shock liver	3 d	Hypotensive, elevated ammonia levels	CRRT, lactulose; 13d	Died
Lung	Kypreos, M. 2021 [49]	Female	68	Somnolent, obtundation	12 d	ammonia level was 226 umol/L	Levofloxacin, azithromycin; 7 d	Recovered
Lung	CharlotteMichel, 2021 [50]	Male	55	Acute respiratory distress, status epilepticus at d5	4 d	Serum ammonia concentration was 661 µg/dL	Azithromycin and doxycycline; 8d	Recovered
Lung	Paparoupa, M.2020 [51]	Female	51	Agitation and was disoriented to place, time and person.	pod	Ammonia387 mmol/l	Doxycycline	Recovered
Lung	Matson, K. M.2019 [52]	Male	62	Lethargic and difficult to arouse	28 d	WBC of 20 k/ul, ammonia level of 196 umol/L	Doxycycline; 10 d	Recovered
Lung	McLaughlin, D. C.2018 [31]	Male	65	Refractory status epilepticus	7 d	ammonia level of 830 µmol/L	Mannitol and 23.4% hypertonic saline, CRRT	Died
Lung	Bharat, A.2015 [8]	Male	44	Acute mental status changes	7 d	Routine blood, urine, and BAL fluid cultures were negative	Azithromycin (500 mg daily for 5 days)	Died
Lung	Wylam, Mark E.2013 [53]	Female	64	Fever to 38.4 °c, and became less alert.	4 d	POD7d ammonia was greater than 704 μmol/L	Ciprofloxacin (400 mg IV twice daily), replaced by cefepime later	Died

**Table 2d.** Summary of review cases Information of the heart transplantation.

Trans organ	Author/year	Gender	Age	Symptom	Time post transplant	Anomaly index	Drug /course	Prognosis
Heart	Sankhesara, D.M. 2022 [24]	Male	63	Hallucinations on D8 and paranoia and reduced GCS on D9	8d	Mildly elevated urea, reatinine, bilirubin & transaminase. Serum ammonia was 549 µmol/L	Moxifloxacin and minocycline; 7d	Recovered
Heart	Leaphart, D. 2021 [54]	Male	55	Diarrhea, anorexia and nausea	5m	Blood ammonia level was 185 µmol/L	Lactulose and azithromycin, CRRT	Recovered
Heart	Schwartz, D.J. 2019 [55]	Female	20	Suprapubic pain, flank pain, and dysuria	18y	Kidney ultrasonography demonstrated new left hydronephrosis	Doxycycline, 100 mg, bid; 25d	Recovered
Heart	Madathil, R. J. 2018 [56]	Male	44	Refractory nausea and, became btunded, progressed to status epilepticus later	3d	Ammonia level >41,320 μmol/L	Azithromycin (eventually changedto levofloxacin) and doxycycline; 40d	Recovered

**Table 2e.** Summary of review cases information of the combined organ transplantation.

Trans organ	Author/year	Gender	Age	Symptom	Time post transplant	Anomaly index	Drug /course	Prognosis
Heart and liver	Shahid, M. 2022 [57]	Male	39	Altered mental status with non-purposeful movements	Immediate	Ammonia level was over 1300 mcg/dL.		Recovered
Kidney and liver	Cannon, C. A. 2020 [5]	Female	53	Mental status rapidly deteriorated	23 d	Electrolytes, blood gas analysis and hepatic function testing were all normal	Doxycycline and levofloxacin	Recovered
Kidney and pancreas	Okumura, Y. 2018 [58]	Male	40	Left-side abdominal pain and had a 38˚C spike like fever	21 d	WBC count of 8. 17 × 109 /L and CRP level of 35.6 mg/L, CT revealed abscesses around the pancreas graft	MINO (100 mg twice a day); 14d	Recovered
Kidney and pancreas	Rohner, P.2004 [59]	Female	33	Fever, diarrhea and abdominal fluid collection	10 d		Doxycycline and ciprofloxacin	Recovered
Kidney and pancreas	Rohner, P.2004 [59]	Female	56	Subfebrile peaks (38 to 38.5 C)		CT showed kidney fluid collection	Doxycycline; 8d	Recovered
Kidney and pancreas	Geissdörfer, W.2001 [60]	Female	43		29d	Leucocyte counts from 13,000/ml to 25,700/ml; CT revealed intra-abdominal abscess	Ciprofloxacin (2*200 mg daily)	Recovered

### 3.4. Data extraction

Case information was gathered by thoroughly reviewing the full text, which included details such as the publication year, patient’s gender, age, history of organ transplantation, onset of UU infection, presenting symptoms, abnormal test findings, administered therapeutic interventions and prognosis. File organization and filtering were performed using Endnote X9 (Clarivate, Philadelphia, PA, USA), and data extraction and sorting were performed using Microsoft Excel 2018 (Microsoft Corporation, Redmond, WA, USA).

## 4. Results

### 4.1. Baseline characteristics of the patients

**Figure 3.**
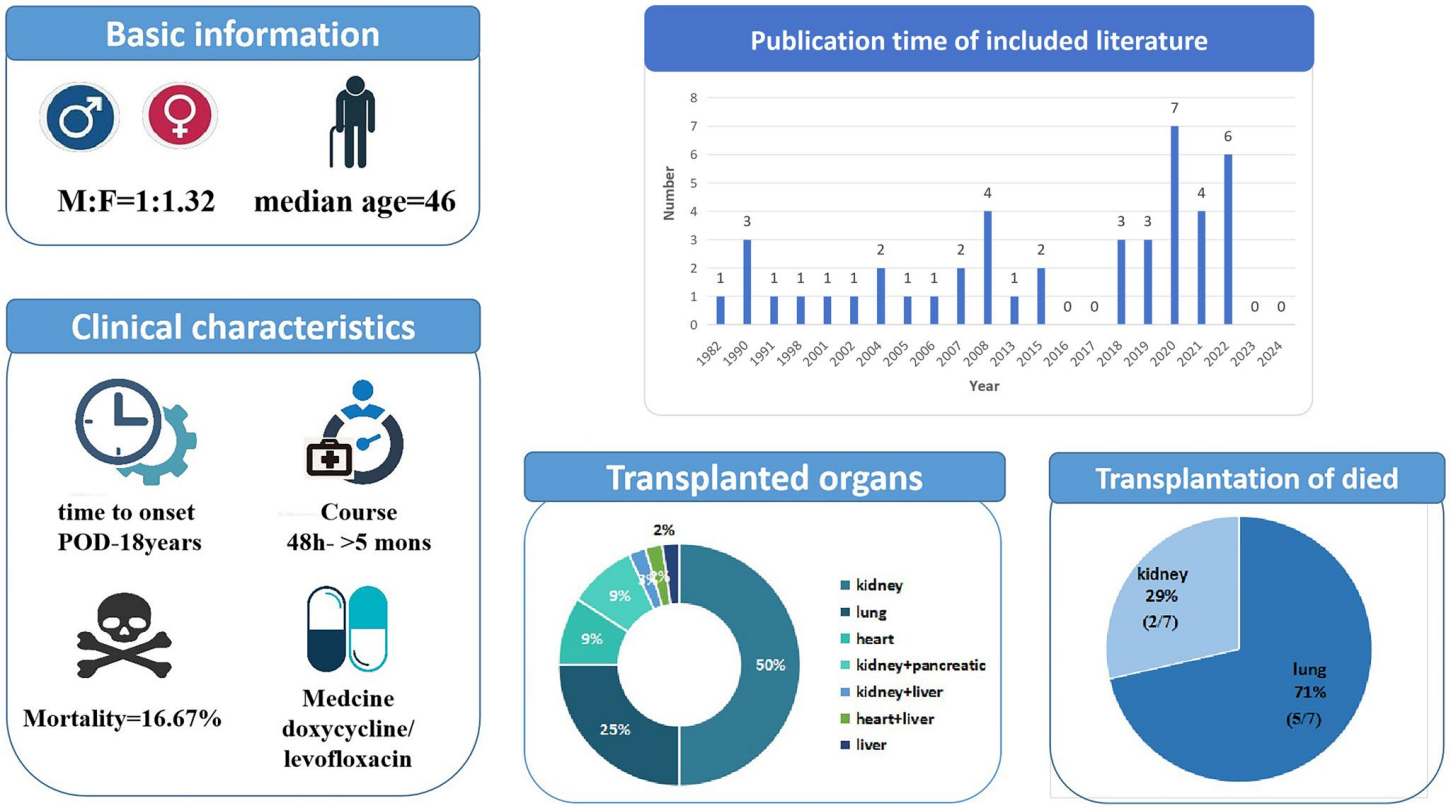
Basic information of this review.

Patients diagnosed with UU infection had an age range of 15–68 years, with a median age of 46 years. Among them, there were 19 men and 25 women, resulting in a man-to-woman ratio of approximately 1:1.32. According to the transplanted organs, there were 22 cases of kidney transplantation, 11 cases of lung transplantation, 4 cases of heart transplantation, 1 case of liver transplantation and 6 cases of multiple organ transplantation (5 cases of kidney and pancreas and 1 case of heart and liver) (see Figure 3). The time of occurrence of *Ureaplasma* infection post-transplantation was as follows: the shortest duration was during the operation (4 cases), whereas the longest duration observed was 18 years post-operation (1 case). Most infections occurred within several days after the operation. Only 5 cases showed an infection occurrence more than 1 year after transplantation, with 72.73% of cases (32 cases) occurring within 1 month post-transplantation.

### 4.2. Clinical characteristics of the patients

The symptoms of *UU* infection in patients after organ transplantation mainly include sudden unexplained disturbance of consciousness and elevated blood ammonia, despite normal liver function indexes. Patients who have undergone renal transplantation may also present with local infection symptoms such as perirenal edema. After the infection, 20 cases showed mental symptoms (restlessness, insanity, disorientation, inattention, seizures, status epilepticus and lethargy), 15 cases had a fever and 12 cases had hydrops or pain in transplanted organs. These symptoms could manifest individually or concurrently. Additionally, 19 cases showed elevated blood ammonia levels, 13 cases had elevated WBC counts, 1 case had mildly elevated creatinine levels and 12 cases had negative findings on blood routine tests, liver function tests and kidney function tests.

### 4.3. Treatment strategy

Before identifying specific pathogenic microorganisms, symptomatic treatments such as hemodialysis or continuous renal replacement therapy (CRRT) were typically administered to reduce ammonia levels. Additionally, lactulose and rifaximin were employed to cleanse the intestinal tract. If leukocyte levels were elevated and CRP or PCT increased, broad-spectrum antibiotics, especially β-lactams, should be prescribed. However, the efficacy of β-lactam antibiotics in controlling UU infections is often limited because UU bacteria lack cell walls, which are the target of β-lactam antibiotics. Therefore, clinical symptoms may not be effectively alleviated with β-lactam antibiotics. Identification methods for UU include culture medium and PCR sequencing. The therapeutic drugs used for this illness include tetracyclines (doxycycline or minocycline), quinolones (ciprofloxacin, ofloxacin/levofloxacin or moxifloxacin) and azithromycin of macrolides. Clinical symptoms can show significant improvement within 24 h of initiating these medications [5].

### 4.4. Clinical characteristics of the deceased patients

Among the 44 patients, 7 patients died, 5 patients received lung transplantation (accounting for 45.5% of the total number of lung transplantation) and 2 patients received kidney transplantation (accounting for 9.1% of the total number of kidney transplantation) (see Table 3). The patients had an age range of 30–65 years, with a man-to-woman ratio of 4:3 and a median age of 44 years. The onset time was 7 days to 3 months after transplantation. Apart from a patient who experienced shock liver, the remaining patients exhibited altered mental states and elevated blood ammonia levels. Despite efforts to lower blood ammonia levels, the patient’s life could not be saved.

**Table 3.** Summary of dead patients infected with *Ureaplasma urealyticum* after transplantation in review.

Trans organ	Author/year	Gender	Age	Symptom	Time post transplant	Anomaly index	Drug /course
Lung	Bharat, A. 2015 [8]	Male	44	Acute mental status changes	7 d	Routine blood, urine, and BAL fluid cultures were negative	Azithromycin (500 mg daily for 5 days)
Lung	Chan, P.G. 2021 [17]	Male	34	Shock liver	3 d	Hypotensive, elevated ammonia levels	CRRT, lactulose; 13d
Lung	McLaughlin, D. C. 2018 [31]	Male	65	Refractory status epilepticus	7 d	Ammonia level of 830 µmol/L	Mannitol and 23.4% hypertonic saline, CRRT
Lung	Shrestha, K. 2022 [48]	Female	30	Decreased responsiveness	7 d	Ammonia levels 353 mmol/l	CRRT, lactulose, polyethylene glycol, levocarnitine, rifaximin, acarbose & sodium benzoate
Kidney	Legouy, C. 2020 [35]	Female	65	Refractory status epilepticus	3 months	Serum ammonia 671 μmol /L	Levofloxacin(500 mg, qd) and doxycyline(200 mg, bid); 4 w
Lung	Wylam, Mark E. 2013 [53]	Female	64	Fever to 38.4 °C, and became less alert.	4 d	POD7d ammonia was greater than 704 μmol/L	Ciprofloxacin (400 mg IV twice daily), replaced by efepime later
Kidney	Kiberenge, R. K. 2015 [38]	Male	35	Nausea, vomiting, and altered mental status	9 d	Ammonia level of 646 μmol/L repeat level 3 h later was 939 μmol/L	CRRT, sodium phenylbutyrate, and sodium benzoate

CRRT: continuous renal replacement therapy.

## 5. Discussion

A case at our hospital presented with unexplained severe neurological signs and hyperammonaemia after kidney transplantation, despite the absence of liver disease, suggesting a possible UU infection, which was later confirmed. This case highlights the potential dangers of UU in patients with immunodeficiency, contrasting with its usual rarity in causing symptomatic infection in individuals with a healthy immune system. Studies have shown that UU rarely induces symptomatic infection in patients with a healthy immune system but may cause disease in patients with immunodeficiency. In patients who undergo organ transplants, graft loss and death may result [5–7]. Patients receiving organ transplantation, such as kidney transplants, have immune system dysfunction due to the use of immunosuppressive drugs, which increases the risk of UU infection. *Ureaplasma urealyticum* can induce hyperammonaemia through the hydrolysis of urea into ammonia, catalyzed by cytoplasmic urease. This process generates energy and typically poses no clinical repercussions in genitourinary infections. However, in disseminated infections, the consequences can be severe: elevated serum ammonia levels prompt the liver to convert it back into urea, whereas urine serves as an energy source for microorganisms, leading to persistent hyperammonemia [8]. The clinical manifestation of hyperammonaemia is severe neurological symptoms, as shown in this case. If it is not diagnosed and treated in time, the consequences can be extremely serious.

Current research and clinical practice have shown that standard microbiological detection methods may not be sufficient for detecting UU infection in patients with immunodeficiency due to the organism’s lack of cell walls. This characteristic prevents visualization under light microscopy with Gram stain and inhibits growth on conventional media. *Ureaplasma urealyticum* is not reported in routine microbiological sample testing, as it can only be isolated with a special medium containing urea. Despite experiencing symptoms such as lumbar discomfort, perirenal abscess or effusion, patients who undergo renal transplantation may present with negative urine culture results, often leading to an oversight in including them in clinical antibiotic treatment plans [9–11]. However, in recent years, due to the continuous promotion and application of second-generation sequencing technology, the detection rate of UU has gradually increased. Its detection in perigraft fluid and blood is more favorable than in urine or secretions [12], and new methods can be used to detect UU and improve the accuracy of diagnosis.

Because of its metabolic characteristics, UU is the main pathogenic microorganism of non-bacterial genitourinary system infection, which can cause renal abscesses and abdominal infection. Renal allograft recipients may have increased susceptibility to UU infection [13]. Genitourinary system *Mycoplasma* infection is more likely to occur in women who undergo transplantation, and about 20%–50% of infections occur in sexually active women [14]. Out of the 22 reviewed patients who underwent renal transplantation, 15 were women, accounting for 68.18% of the total. Gerber L. et al. proposed that anti-infection treatment covering UU should be carried out promptly when the following symptoms occur: symptomatic urinary tract infection with negative standard urine culture, including pyelonephritis; symptoms of urethritis such as urethral discharge, pruritus or dysuria; graft dysfunction in patients with aseptic leucocytosis; early postoperative deep wound infection (with inconclusive standard culture results of drainage fluid); unexplained hyperammonemia [15,16]. The drainage fluid and blood from the infected site were collected for NGS detection. Given the potential severity of UU infection, understanding its drug resistance is a key part of ensuring an effective treatment strategy.

The results of drug resistance analysis of UU in different studies were significantly different. A French study showed that the resistance rate of UU to tetracycline was 7.5% and that its resistance rates to levofloxacin and moxifloxacin were 1.2 and 0.1%, respectively. Moreover, the drug resistance rate of *Mycoplasma hominis* to tetracycline was 14.8%, and its resistance rates to levofloxacin and moxifloxacin were 2.7% and 1.6%, respectively [15]. Furthermore, an Italian study revealed a significant level of drug resistance in UU to quinolones, particularly ciprofloxacin (77%) [15]. In addition, in about 90% of cases, *M. hominis* strains were insensitive to azithromycin and roxithromycin [17]. Therefore, the combination of quinolones, tetracyclines and azithromycin is often selected for the treatment of UU [18]. In addition to drug treatment, continuous hemofiltration can significantly improve the survival rate of patients infected with UU and HS [18].

Azithromycin is a macrolide antibiotic that interferes with protein synthesis by binding to the subunits of 50S subunits of susceptible microorganisms. In addition to its good bactericidal effect on common bacteria, it also effectively kills various atypical pathogens such as *Mycoplasma*, *Chlamydia* and *Spirochete*. In the reported case, the patient experienced adverse reactions, including arrhythmia, after using levofloxacin. Consequently, azithromycin was chosen as an alternative treatment. The incidence of UU infection decreased from 27.5% to 9.7% (*p* = 0.01) when the graft donor was empirically treated with quinolones or azithromycin. The median duration of donor antibacterial treatment was only 1.7 days [19]. Screening donors for UU and incorporating drugs targeting UU into empirical anti-infection protocols for post-transplantation patients may reduce the occurrence of mental symptoms and sequelae associated with hyperammonaemia following severe UU infection [19–21].

Patients who undergo lung transplantation may suffer from hyperammonaemia due to UU infection, and these patients have a high mortality rate (60%–70%) [16,22]. The total number of patients who underwent lung transplantation included in this review was small, and the mortality rate was 45.45%, which was the highest among different organ transplants. Compared with other solid organ transplant recipients, lung transplant recipients generally experience higher levels of immunosuppression, more challenging surgical procedures, longer periods of ventilation support and more complex regimens for both induction and maintenance of immunosuppressive therapy [23]. Infection with UU or *Ureaplasma parvum* is associated with hyperammonemia [10], and patients with hyperammonaemia mainly present nervous system symptoms, often accompanied by brain edema as a typical imaging change. Furthermore, HS caused by pathological brain changes includes brain edema, astrocyte swelling, white matter damage and internal cellular reactions caused by brainstem hernia. Ammonia exposure changes the kinase pathway and the expression of potassium channels and aquaporins. Amino acids are disordered in the brain and change the neural transmission system, including glutamate and serotonin systems [24]. Bharat et al. described that four lung transplant recipients developed hyperammonaemia with brain edema within a week of transplantation. The condition was not responsive to various treatment strategies, including lactulose, CRRT, L-carnitine and rifaximin. Hypertonic saline and mannitol can prevent the worsening of astrocyte swelling by maintaining serum osmolality and has similar efficacies in reducing intracranial pressure [25]. However, the rebound increase in intracranial pressure was significantly higher with mannitol than hypertonic saline [26,27]. Mannitol not only temporarily increases plasma osmolality leading to an increase in extracellular fluid and thus improves renal circulation, but also has the effect of renal vasodilation and an increase in renal plasma flow [28]. In addition, mannitol was beneficial in reducing primary acute tubular necrosis of the transplanted kidney [29]. We chose mannitol, considering that this patient had undergone a kidney transplant and that he had severe cerebral edema and that he needed to treat cerebral edema in a short period of time without kidney injury. The death of the four patients was not prevented [8]. The paraffin-embedded lung tissues of all patients during autopsy were detected by PCR, and the results were positive for UU. In addition, the patients’ blood and bronchoalveolar lavage (BAL) PCR were found positive. In contrast, Bharat et al. found that the PCR (blood and BAL) of 20 lung transplant recipients without HS were negative for UU, *Ureaplasma parvum* and *M. hominis*. To prospectively verify this possible relationship, Bharat et al. treated 2 patients with refractory HS and positive test results post-lung transplantation with levofloxacin/azithromycin and levofloxacin, respectively. In both cases, hyperammonaemia disappeared after medication, and the patients recovered completely [30]. Wang et al. [23] observed in the mouse model infected with UU that the use of immunosuppressants after lung transplantation is an important cause of hyperammonaemia. In addition, the lungs of mice with hyperammonaemia had no or only mild pneumonia symptoms, indicating that the imaging examination of the lungs had no guiding significance for patients who undergo lung transplantation to be infected with UU [31].

## 6. Conclusion

There is a possibility of severe UU infection in patients with immunodeficiency after organ transplantation. Additionally, UU infection should be considered in patients presenting with severe neurological symptoms and hyperammonaemia in the absence of liver disease. Prompt treatment is crucial to improve prognosis, potentially reducing severe nerve damage, organ dysfunction and mortality.

## Supplementary Material

Fig2.jpg

Fig3.jpg

Publication time of included literature.docx

Fig1.jpg
