# Supplementary figures and images for: Ureaplasma urealyticum infection following organ transplantation: a case report and narrative review
Source: Ren Fail. 2024 Aug 27;46(2):2395466. doi: 10.1080/0886022X.2024.2395466 (PMC11360648; doi:10.1080/0886022X.2024.2395466)

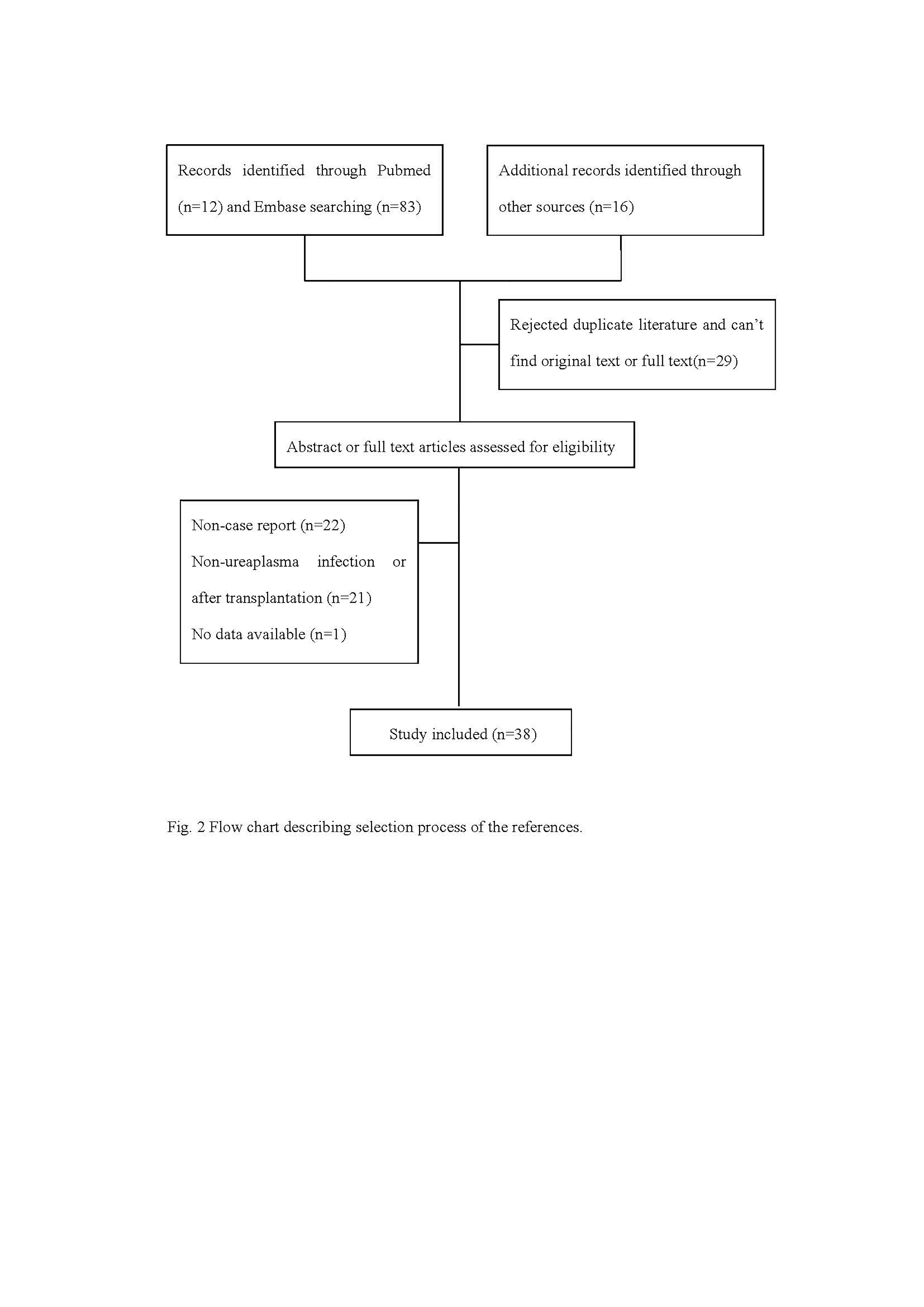

Supplement: Fig2.jpg [file IRNF_A_2395466_SM0855.jpg]

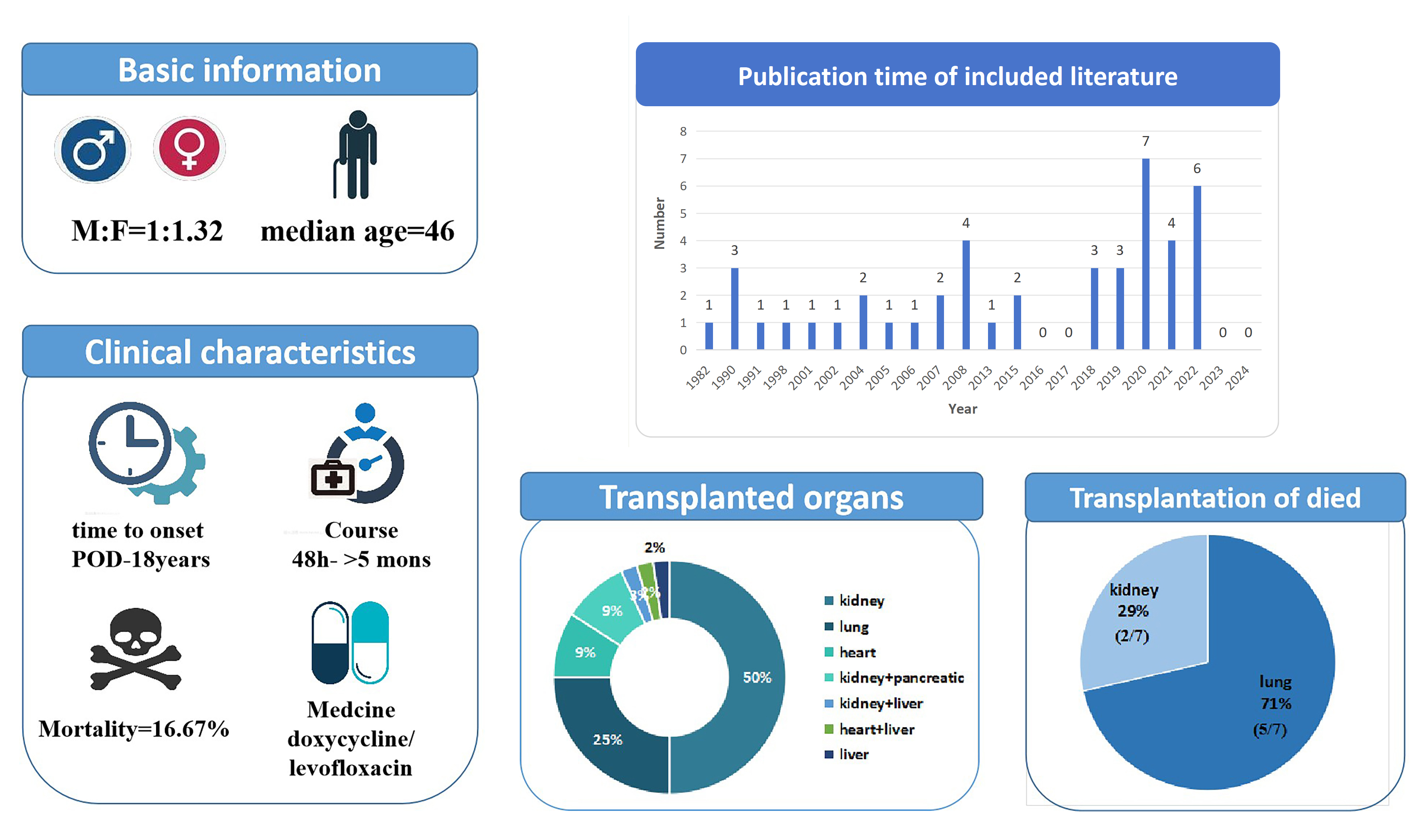

Supplement: Fig3.jpg [file IRNF_A_2395466_SM0854.jpg]

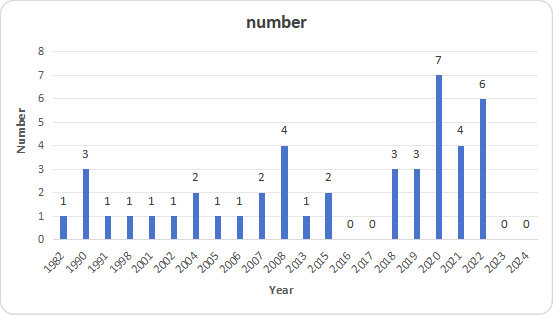


**Publication time of included literature**

Supplement: Publication time of included literature.docx [file IRNF_A_2395466_SM0853.docx]

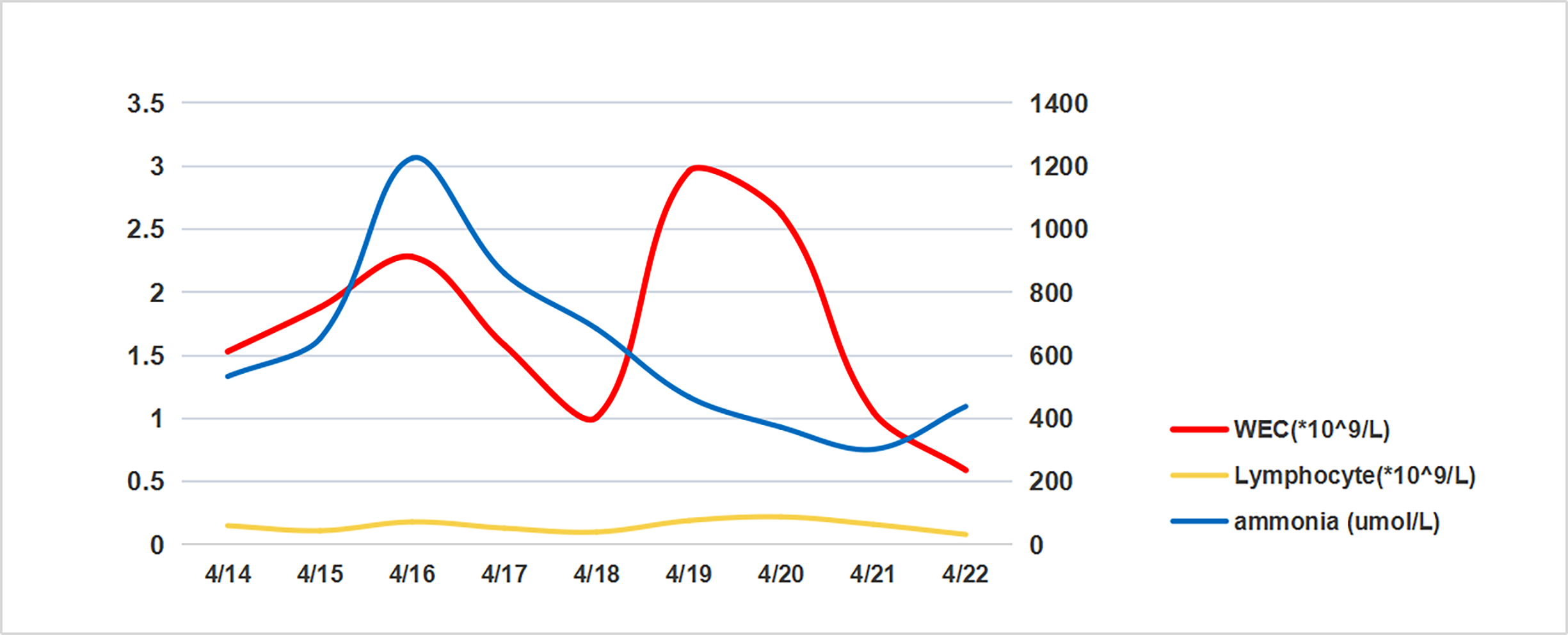

Supplement: Fig1.jpg [file IRNF_A_2395466_SM0852.jpg]
